# Single-Ion Behavior in New 2-D and 3-D Gadolinium 4f^7^ Materials: CsGd(SO_4_)_2_ and Cs[Gd(H_2_O)_3_(SO_4_)_2_]·H_2_O

**DOI:** 10.1021/acsorginorgau.2c00031

**Published:** 2022-09-04

**Authors:** Ebube E. Oyeka, Thao T. Tran

**Affiliations:** Department of Chemistry, Clemson University, Clemson, South Carolina 29634, United States

**Keywords:** gadolinium, single ion, paramagnetic property, structural dimensionality, Brillouin function

## Abstract

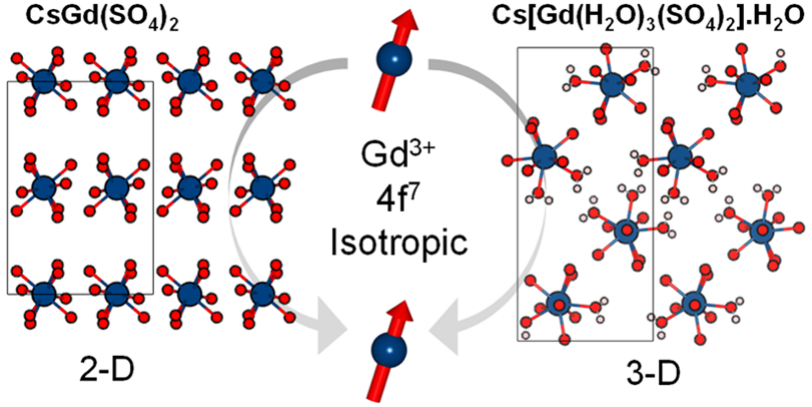

The recent creation of 4f^7^ gadolinium materials has enabled vital studies of the free-ion properties of the Gd(III) cations. While the ^8^*S* ground state in a trivalent Gd compound is, in principle, isotropic, it has been demonstrated that there is a residual orbital angular momentum affected by the crystal field and structural distortion in certain systems. By exploiting the atomistic control innate to material growth, we address a fundamental question of how the isotropic nature of Gd(III) is preserved in different dimensionalities of crystal structures. To achieve this, we designed two new trivalent Gd materials possessing two structurally distinct features, a 2-D CsGd(SO_4_)_2_ and a 3-D Cs[Gd(H_2_O)_3_(SO_4_)_2_]·H_2_O. The tunability of the structural dimension is facilitated by O–H---O hydrogen bonds. The structural divergence between the two compounds allows us to investigate each material individually and make a comparison between them regarding their physical properties as a function of lattice dimension. Our results demonstrate that structural dimensions have a negligible effect on the single-ion behavior of the materials. Magnetization measurements for the Gd(III) complexes yielded paramagnetic states with the isotropic spin-only nature. Specific heat data suggest that there is a lack of magnetic phase transition down to *T* = 1.8 K, and coupled lattice vibrations in the materials are attributable to strong covalent bonding characters of the (SO_4_)^2–^ and H_2_O ligands. This work offers a pathway for retaining the single-ion property of Gd(III) while constructing the large spin magnetic moment *S* = 7/2 in large-scale extended frameworks.

## Introduction

Lanthanide (III) materials have been of particular interest in new physical phenomena owing to their capability to exhibit strong correlation physics in the presence of large spin–orbit coupling.^1−19^ Among the trivalent ions of the lanthanides, gadolinium(III) is unique in that its ground state is, in principle, isotropic (^8^*S*_7/2_). The magnetic moment of the Gd^3+^ cation originates from the spin *S* = 7/2, while the orbital angular momentum *L* is completely quenched.^20,21^ This large spin magnetic moment of Gd^3+^ materials renders them appealing systems for studies in magnetism and low-temperature magnetocalorics.^20,22−28^ In addition, Gd compounds come closest to the ideal model wherein the Gd^3+^ cations are completely isolated from each other and experience only a weak static field generated by the other ions. This is a prerequisite for other useful functionalities and spectroscopic investigations.^15−21^ Nevertheless, structural distortion and crystal fields have been proved to influence the orbital angular momentum of some Gd^3+^ materials such as Mg_2_Gd_3_Sb_3_O_14_, Gd_2_Ti_2_O_7_, and SrGd_2_O_4_, disrupting the single-ion property of these systems.^22−24,29^

While these studies paved the way for exploring new capabilities of Gd^3+^ materials, the observed magnetic anisotropy recalibrates the expectation of preserving the isotropic integrity of the 4f^7^ electronic structure in extended frameworks. This is, in part, attributable to insufficient characterization of magnetism and thermodynamics of the Gd^3+^ isotropy while incorporating the *S* = 7/2 in different dimensionalities of crystal lattice. Understanding of the degree of the isotropic character of Gd(III) is crucial in guiding design considerations of single-ion physics, qubits, and magnetocaloric coolants based on fundamental variations in the lanthanide–anion bonding as a function of lattice dimension. To address this challenge, we created two new Gd^3+^ materials exhibiting distinct structural dimensionalities: 2-D CsGd(SO_4_)_2_ and 3-D Cs[Gd(H_2_O)_3_(SO_4_)_2_]·H_2_O. The dimensional extension of the structures is enabled by O–H---O hydrogen bonds. Herein, we describe the synthesis, structural analysis, spectroscopy, magnetic, and heat capacity investigation of these two Gd materials.

## Experimental Section

### Reagents

For this work we used the following reagents: thiophene-2-carbonyl chloride (ACROS Organics), aniline (ACROS Organics), KSeCN (ACROS Organics), Gd(NO_3_)_3_·6H_2_O (ACROS Organics), CsCl (VWR), HNO_3_ (Fisher, 67%), H_2_SO_4_ (Fisher, 98%), acetone (ACROS Organics), ethanol (Fisher), dichloromethane (ACROS Organics).

### Synthesis of C_12_H_10_N_2_OSSe (TAS)

Acetone solutions containing thiophene-2-carbonyl chloride (0.01 mol, 1.47 g, 30 mL) and KSeCN (0.01 mol, 1.44 g, 30 mL) were mixed and stirred at room temperature for 30 min. Suspended solid was removed by filtration. Acetone solution containing aniline (0.01 mol, 0.93 g) was added to the filtrate and stirred at 60 °C for 1 h. TAS was isolated from the mother liquor as yellow needle crystals, washed with water and ethanol, and dried in air. Recrystallization and purification were done using 1:2 ethanol/dichloromethane mixture (yield, 1.9 g, 63.5% based on KSeCN); mp 143 °C; ^1^H NMR (500 MHz, CDCl_3_) δ 12.83 (s, 1H, NH), δ 9.32 (s, 1H, NH), δ 7.78–7.79 (m, 2H, CH), δ 7.70–7.72 (d, 2H, CH), δ 7.45–7.48 (t, 2H, CH), δ 7.35–7.38 (t, 1H, CH), δ 7.23–7.25 (t, 1H, CH); ^13^C NMR (500 MHz, CDCl_3_) δ 179.7 (C=O), 161.1 (C=Se), 138.4–124.7 (Ar–C); MS (MALDI-TOF) *m*/*z* calcd for C_12_H_10_N_2_OSSe [M]^+^ 309.97, *m*/*z* found 309.94; IR ν (cm^–1^) 3264 (νN–H), 1519 (νC=O), 632 (νC=Se); UV/vis (nm) 294 (π* ← n, C=O, ε_λmax_ = 23651 dm^3^ mol^–1^ cm^–1^), 336 (π* ← n, C=Se).

### Synthesis of CsGd(SO_4_)_2_

Gd(NO_3_)_3_·6H_2_O (2 mmol, 0.903 g) and CsCl (2 mmol, 0.337 g) were dissolved in 3.6 M H_2_SO_4_ (10 mL) in a 23 mL PTFE-lined autoclave. The autoclave was heated at 200 °C for 60 h and cooled slowly to 25 °C at the rate of 5 °C/h. CsGd(SO_4_)_2_ was isolated as plate-shaped colorless crystals by filtration, washed with deionized water, and dried (yield, 0.36 g, 37% based on Gd).

### Synthesis of Cs[Gd(H_2_O)_3_(SO_4_)_2_]·H_2_O

Gd(NO_3_)_3_·6H_2_O (2 mmol, 0.903 g) and CsCl (2 mmol, 0.337 g) were dissolved in 4 M HNO_3_ (10 mL) in a 23 mL PTFE-lined autoclave, and TAS (2 mmol, 0.62 g) was added. The autoclave was heated at 200 °C for 60 h and cooled slowly to 25 °C at the rate of 5 °C/h. A pale green solution was obtained and was left to evaporate slowly. Cs[Gd(H_2_O)_3_(SO_4_)_2_]·H_2_O was isolated as block-shaped colorless crystals from the solution after 1 week. These crystals were gently washed with deionized water and dried (yield, 0.32 g, 58% based on Gd).

### Single-Crystal X-ray Diffraction

Single-crystal crystallographic data of TAS (at 100 K) (Figures S1 and S2) were collected using a Bruker D8 Venture diffractometer with Mo Kα radiation (λ = 0.71073 Å) and a Photon 2 detector. Single-crystal data of CsGd(SO_4_)_2_ and Cs[Gd(H_2_O)_3_(SO_4_)_2_]·H_2_O were collected on a Bruker DUO platform diffractometer equipped with a 4K CCD APEX II detector using Mo Kα radiation (λ = 0.71073 Å) and a Photon 100 detector. Data processing (SAINT) and scaling (SADABS) were performed using the Apex4 software system. The structures were solved by intrinsic phasing (SHELXT) and refined by full-matrix least-squares techniques on *F*^2^ (SHELXL) using the SHELXTL software.^30^ All atoms were refined anisotropically. Crystal structures were viewed with VESTA.^31^

### Powder X-ray Diffraction

Powder X-ray diffraction (PXRD) data on CsGd(SO_4_)_2_ were collected using a Rigaku Ultima IV in the 2θ range of 5–90° at a 0.2°/min scan rate. PXRD measurements on Cs[Gd(H_2_O)_3_(SO_4_)_2_]·H_2_O were performed using a Bruker D2 Phaser diffractometer with a LynxEye-XE-T detector. Data were collected in the 2θ range of 5–120° at 0.64°/min. Rietveld refinement of XRD patterns was performed using TOPAS Academic V6.

### NMR

^1^H and ^13^C NMR spectra of TAS were obtained using a 500 MHz Bruker NMR spectrometer in CDCl_3_ solution (Figures S3 and S4).

### Mass Spectroscopy

Mass spectrum of TAS was obtained from Q-TOF using matrix-assisted laser desorption/ionization (MALDI) (Figure S5).

### Infrared Spectroscopy

Attenuated total reflection Fourier transform infrared (ATR-FTIR) spectra for TAS, CsGd(SO_4_), and Cs[Gd(H_2_O)_3_(SO_4_)_2_]·H_2_O were collected separately using a Shimadzu IR Affinity-1S in 400–4000 cm^–1^ frequency range (Figure S6).

### UV–Vis–NIR Spectroscopy

UV–vis–NIR spectra for TAS, CsGd(SO_4_)_2_, and Cs[Gd(H_2_O)_3_(SO_4_)_2_]·H_2_O were measured using an Agilent Cary UV–vis (NIR) 7000 spectrophotometer with universal measurement accessories. A mixture of approximately 20 mg of each sample and 60 mg of PTFE was pelletized and used for the measurement (Figures S7 and S9).

### Thermal Analysis

Thermogravimetric analysis (TGA) and differential scanning calorimetry (DSC) measurements were performed using a TA SDT Q600 Instrument. Approximately 10 mg of each compound was separately placed in an alumina crucible and heated at a rate of 20 °C/min from room temperature to 1000 °C under flowing nitrogen (flow rate: 100 mL/min) (Figures S8 and S10).

### Magnetization and Specific Heat

DC magnetization measurements on CsGd(SO_4_)_2_ and Cs[Gd(H_2_O)_3_(SO_4_)_2_]·H_2_O powders were performed with the vibrating sample magnetometer (VSM) option of the Quantum Design physical properties measurement system (PPMS). Heat capacity was measured on the single crystals using the PPMS, employing the semiadiabatic pulse technique from *T* = 2 to 300 K (Figures S11 and S12).

## Results and Discussion

### Characterization of C_12_H_10_N_2_OSSe (TAS)

The molecular structure of TAS was determined by single-crystal X-ray diffraction and spectroscopic techniques including ^1^H and ^13^C NMR spectroscopy, UV–vis, IR, and mass spectroscopy. Figure S1 shows the crystal structure of TAS, and crystallographic refinement data are presented in Table S1. TAS crystallizes in the monoclinic space group *P*2_1_/*n* with four molecules in the unit cell. The molecule comprises three structural features: a thiophene ring, a phenyl ring, and an acylselenourea moiety. Further discussion on the crystal structure and spectroscopic characterization of TAS is presented in the Supporting Information (Figures S2–S4). The chemical composition of TAS (C_12_H_10_N_2_OSSe) is consistent with that obtained by mass spectroscopy (Figure S5). The peak at *m*/*z* = 309.94 corresponds to the mass of the parent compound (C_12_H_10_N_2_OSSe)^+^. We also detected fragments of the protonated species (C_12_H_11_N_2_OSSe)^+^ at the base peak (*m*/*z* = 310.93). TAS melts incongruently at *T* = 143 °C and then decomposes at *T* = 174 °C as determined by the TG/DSC measurement (Figure S8).

### Crystal Structure of CsGd(SO_4_)_2_ and Cs[Gd(H_2_O)_3_(SO_4_)_2_]·H_2_O

**Figure 1:**
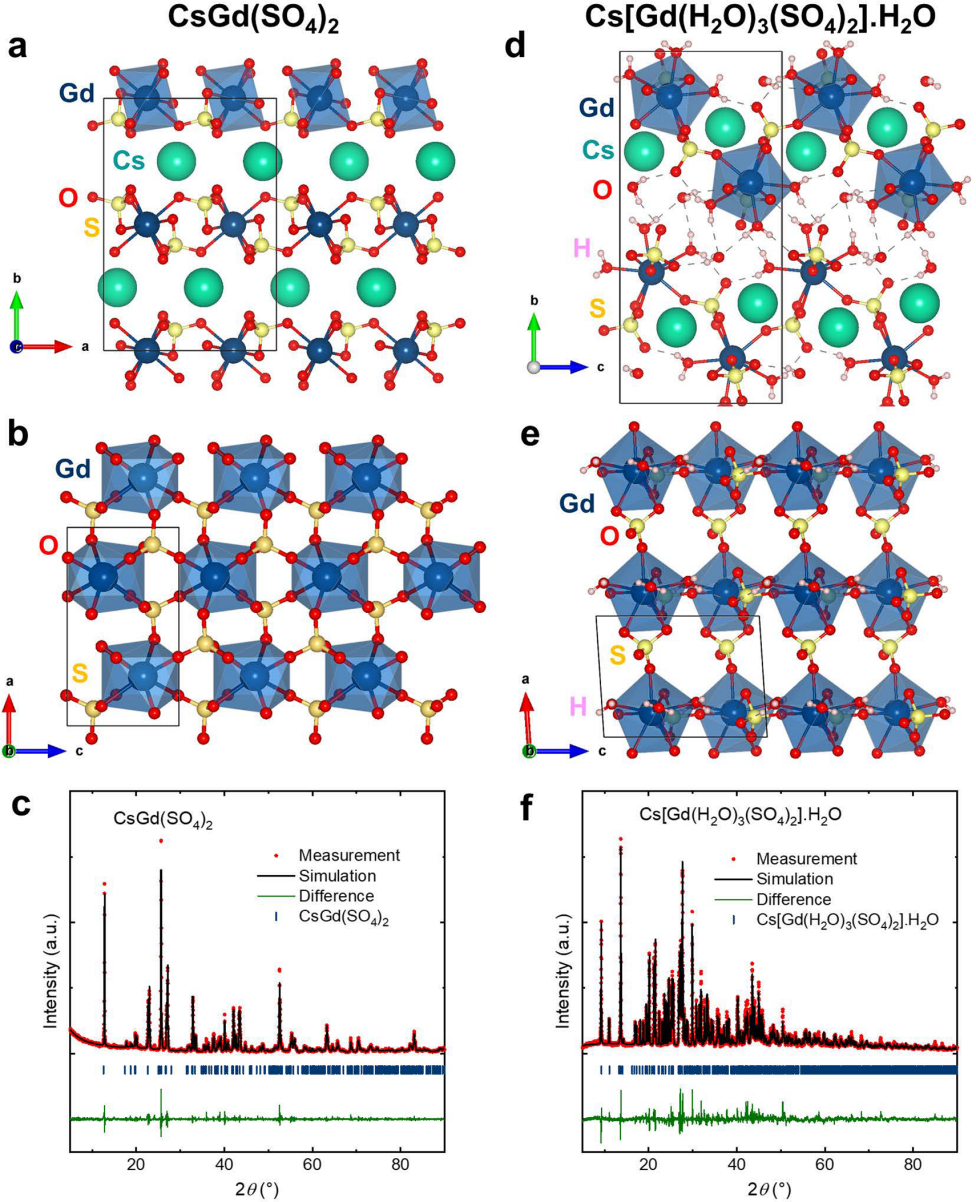
(a,b) Crystal structure of CsGd(SO_4_)_2_ featuring a 2-D structure consisting of [Gd(SO_4_)_2_]^−^ layers separated by Cs^+^ cations. (c) Rietveld refinement of PXRD data for CsGd(SO_4_)_2_. (d,e) Crystal structure of Cs[Gd(H_2_O)_3_(SO_4_)_2_]·H_2_O consisting of a 3-D framework of GdO_6_(H_2_O)_3_ distorted capped square antiprisms connected through (SO_4_)^2–^ tetrahedra; the Cs^+^ cation occupies the empty space formed by the framework. (f) Rietveld refinement of PXRD data for Cs[Gd(H_2_O)_3_(SO_4_)_2_]·H_2_O. There is an excellent agreement between the PXRD and single-crystal XRD data, and no impurity was observed.

The crystal structures of CsGd(SO_4_)_2_ and Cs[Gd(H_2_O)_3_(SO_4_)_2_]·H_2_O were determined by single-crystal XRD (Figure 1a,b,d,e and Table 1). The crystal structures obtained from Rietveld refinements of PXRD data are in excellent agreement with those deduced from single-crystal XRD (Figure 1c,f).

**Table 1:** Crystallographic Data of CsGd(SO_4_)_2_ and Cs[Gd(H_2_O)_3_(SO_4_)_2_]·H_2_O Obtained by Single-Crystal XRD

	CsGd(SO_4_)_2_	Cs[Gd(H_2_O)_3_(SO_4_)_2_]·H_2_O
formula	CsGdO_8_S_2_	CsGdH_8_O_12_S_2_
*M* (g mol^–1^)	482.28	554.34
*T* (K)	297(2)	297(2)
crystal dimension (mm)	0.23 × 0.18 × 0.07	0.38 × 0.26 × 0.07
X-ray radiation	Mo Kα	Mo Kα
λ (Å)	0.71073	0.71073
crystal system	orthorhombic	monoclinic
space group	*Pnna*	*P*2_1_/*c*
*Z*	4	4
*a* (Å)	9.540(4)	6.5382(1)
*b* (Å)	13.933(5)	19.0619(4)
*c* (Å)	5.370(2)	8.8218(2)
α (deg)	90	90
β (deg)	90	93.850(1)
γ (deg)	90	90
*V*(Å^3^)	713.8(5)	1096.98(4)
ρ_calc_ (g cm^–1^)	4.488	3.357
no. of reflections	519	2741
no. of parameters	56	177
μ (mm^–1^)	14.909	9.750
2θ_max_ (deg)	56.7	56.7
GOF	1.322	1.279
*R*(*F*)^a^	0.0197	0.0208
*R*(*F*_w_^2^)^b^	0.0507	0.0469

a *R*(*F*) = ∑∥*F*_o_| – |*F*_o_^2^∥/∑|*F*_o_|.

b *R*_*w*_(*F*_o_^2^) = [∑*w*(*F*_o_^2^ – *F*_o_^2^)^2^/∑*w*(*F*_o_^2^)^2^]^1/2^.

CsGd(SO_4_)_2_ crystallizes in the centrosymmetric orthorhombic space group *Pnna*. The crystal structure of the Gd material features a 2-D structure consisting of [Gd(SO_4_)_2_]^−^ layers separated by the Cs^+^ cations. Each Gd^3+^ cation is bonded to eight oxygen atoms from the (SO_4_)^2–^ groups, forming an eight-coordinate geometry that can be described as a distorted square antiprism. Gd–O bond distances range from 2.374(4) to 2.470(4) Å. The top basal plane of the square antiprism is rotated from the plane below by ∼29–43°, which deviates from the distortion angle for an ideal square antiprism (45°). The Gd^3+^ cations form a 2-D distorted triangular sublattice with the Gd–Gd distances ranging from 5.1366(3) to 5.8952(5) Å.

**Figure 2:**
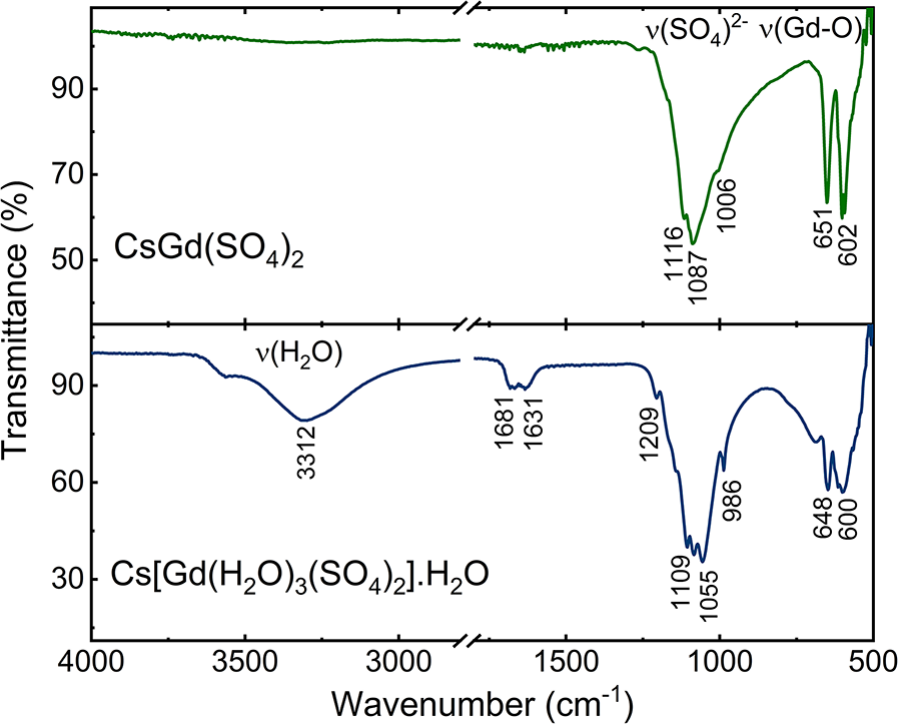
ATR-FTIR spectra of CsGd(SO_4_)_2_ and Cs[Gd(H_2_O)_3_(SO_4_)_2_]·H_2_O.

The (SO_4_)^2–^ anion comprises S^6+^ bonded to four oxygen atoms with S–O bond lengths ranging from 1.477(4) to 1.497(4) Å. The local structure of (SO_4_)^2–^ was confirmed to be the pseudo-*T*_*d*_ point group, Γ_vib_ = 3*T*_2_, as evidenced by the three peaks between 900 and 1200 cm^–1^ in the IR spectra (Figure 2).

Cs[Gd(H_2_O)_3_(SO_4_)_2_]·H_2_O crystallizes in the centrosymmetric monoclinic space group *P*2_1_/*c*. The crystal structure of the Gd hydrate compound features a 3-D framework of GdO_6_(H_2_O)_3_ distorted capped square antiprisms linked through (SO_4_)^2–^ groups. The Gd^3+^ cation in GdO_6_(H_2_O)_3_ is bonded to six oxygen atoms from the (SO_4_)^2–^ groups and three from the H_2_O ligands in a distorted capped square antiprism. Gd–O bond distances range from 2.363(3) to 2.555(3) Å. The (SO_4_)^2–^ anion contains S with the formal oxidation state of +6, bonded to four oxygen atoms in a pseudo-tetrahedral geometry. S–O covalent bond distances range from 1.450(3) to 1.497(3) Å. In contrast to the 2-D structure of the Gd compound, the 3-D network of the Gd hydrate material is formed by Gd–OH_2_---O and S–O---H_2_O hydrogen bonds. The Gd–Gd distances range from 6.4306(6) to 6.53820(11) Å. We confirmed the symmetry and local structures of the building blocks of Cs[Gd(H_2_O)_3_(SO_4_)_2_]·H_2_O by performing IR measurements. The broad bands corresponding to the characteristic O–H stretching vibration of the H_2_O ligand occur at ∼3313 cm^–1^, while the H–O–H bending vibration modes are at ∼1631 and 1631 cm^–1^. These three absorption bands are consistent with the *C*_2*v*_ point group of H_2_O, Γ_vib_ = 2A_1_ + B_1_. The (SO_4_)^2–^ tetrahedron is confirmed to have an approximate *T*_*d*_ point symmetry, Γ_vib_ = 3T_2_. Each inequivalent (SO_4_)^2–^ tetrahedron contributes three peaks, resulting in six peaks between 900 and 1200 cm^–1^ in the IR spectra (Figure 2).

### UV–Vis–NIR Spectroscopy

To probe the electronic transitions associated with the Gd^3+^ cation (f^7^, *S* = 7/2, *L* = 0), we measured UV–vis–NIR reflectance spectra for CsGd(SO_4_)_2_ and Cs[Gd(H_2_O)_3_(SO_4_)_2_]·H_2_O from λ = 390 to 2500 nm (*h*ν ≈ 0.5–3.2 eV). The data are expressed as the Kubelka–Munk function *F*(*R*) versus *h*ν (Figure S9).^32^ Both Gd compounds show no absorption band within the excitation energy window over which the measurements were performed. This behavior is consistent with other Gd^3+^ systems.^33,34^ The ground state of Gd^3+^ is ^8^*S*_7/2_, and the energy gap between the ground state and the next excited state (^6^*P*_7/2_) is close to 5 eV.^33,34^ In addition, there is no spin-allowed transition between the ground state and the excited states. This explains the lack of electronic transition in the UV–vis–NIR data, confirming the ^8^*S*_7/2_ ground state for both Gd materials.

### Thermogravimetric Analysis

The thermal behavior of CsGd(SO_4_)_2_ and Cs[Gd(H_2_O)_3_(SO_4_)_2_]·H_2_O was characterized by TGA and DSC under a nitrogen atmosphere (Figure S10). The decomposition of CsGd(SO_4_)_2_ occurs at 377 °C corresponding to the loss SO_2_. The experimental weight loss (13.24%) is in excellent agreement with the calculated weight loss (13.27%). The endothermic peak in the heating curve is consistent with the decomposition of the Gd compound. In contrast to CsGd(SO_4_)_2_, Cs[Gd(H_2_O)_3_(SO_4_)_2_]·H_2_O has relatively low thermal stability. The Gd hydrate material begins to decompose at 55 °C. The reduction in mass in the temperature range of 55 °C – 265 °C are likely attributable to the loss of 4 H_2_O molecules. The experimental weight loss (13.1%) is consistent with the calculated weight loss (13.0%).

### Magnetization

**Figure 3:**
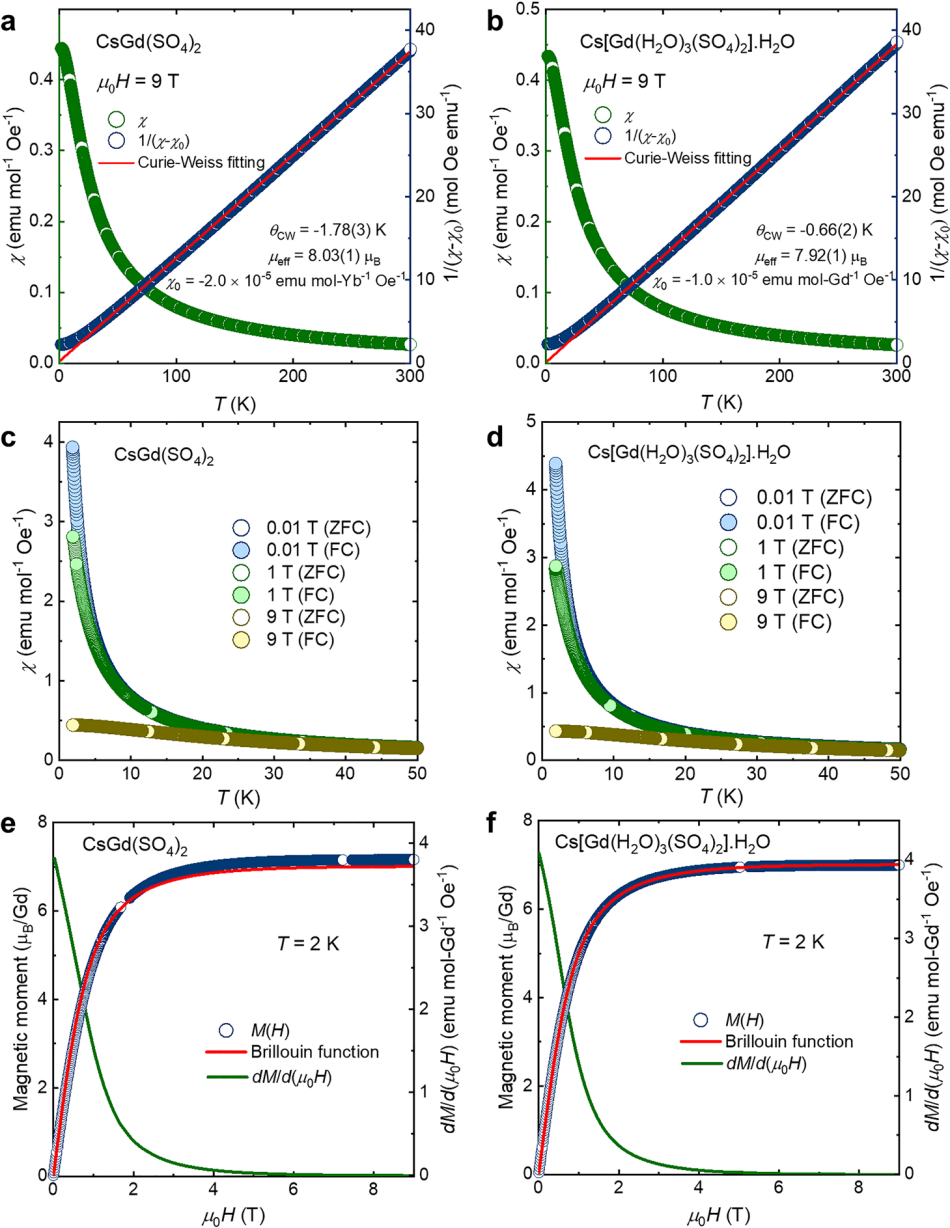
CsGd(SO_4_)_2_ and Cs[Gd(H_2_O)_3_(SO_4_)_2_]·H_2_O. (a,b) DC magnetic susceptibility (χ) from *T* = 2 to 300 K at μ_0_*H* = 9 T; (dark blue) Curie–Weiss fit of 1/(χ – χ_0_) versus *T*. (c,d) Zero-field-cooled and field-cooled magnetic susceptibility at different magnetic fields. (e,f) *M*(*H*) data in μ_B_ at *T* = 2 K; Brillouin function for *J* = 7/2 and *T* = 2 K; d*M*/d*H* curve showing no critical field.

To evaluate the spin state of the Gd^3+^ cation in CsGd(SO_4_)_2_ and Cs[Gd(H_2_O)_3_(SO_4_)_2_]·H_2_O and determine the magnetic properties of the materials, we performed temperature-dependent magnetization at μ_0_*H* = 9 T and field-dependent magnetization at *T* = 2 K (Figure 3). The magnetic susceptibility data of these materials follow the Curie–Weiss law over a wide temperature range of 30 K ≤ *T* ≤ 300 K (eq 1):^35^
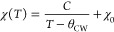
1where *C* is the Curie constant, θ_CW_ is the Curie–Weiss temperature, and χ_0_ is the temperature-independent contribution to the susceptibility, which includes the small diamagnetic signals of the electron core and the sample holder.^36^ The effective magnetic moment μ_eff_ per Gd^3+^ cation was estimated using eq 2:
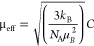
2where *N*_A_ is the Avogadro number and *k*_B_ is the Boltzmann constant.

The effective magnetic moments μ_eff_ per Gd^3+^ cation obtained for CsGd(SO_4_)_2_ and Cs[Gd(H_2_O)_3_(SO_4_)_2_]·H_2_O are 8.03(1)μ_B_ and 7.92(1)μ_B_, respectively. These values are very close to the effective magnetic moment expected for a spin-only system (μ_eff_ = 7.94 μ_B_) (Figure 3a,b). The Curie–Weiss temperature value was obtained to be θ_CW_ = −1.78(3) K for the Gd compound and θ_CW_ = −0.66(2) K for the Gd hydrate material, indicating very weak to negligible interaction between the Gd^3+^ spins. These values are similar to those reported for other relevant Gd^3+^ compounds such as Sr_2_GdSbO_6_ (θ_CW_ = −0.1(4) K), KBaGd(BO_3_)_2_ (θ_CW_ = −0.78 K), Ba_2_GdSbO_6_ (θ_CW_ = −1.2(2) K), and Gd_3_Sb_3_Zn_2_O_14_ (θ_CW_ = −2.32 K).^24,37−39^ Zero-field-cooled and field-cooled magnetization data (Figure 3c,d) measured at different magnetic fields show no bifurcation, confirming no magnetic phase transition down to *T* = 2 K. The Brillouin function describes the magnetic moment of Gd^3+^ noninteracting spins at *T* = 2 K (eqs 3–5).^40^
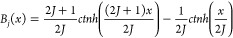
3
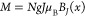
4
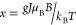
5*B*_*J*_(*x*) is the Brillouin function, *M* is the magnetization, μ_B_ is the Bohr magneton, *N* is the gas constant, *B* is the magnetic field, and *k*_B_ is the Boltzmann constant.

Overall, the *M*(*H*) curves of both Gd compounds overlap with this Brillouin model, suggesting a paramagnetic ground state and magnetic isotropy (Figure 3e,f). The *M*(*H*) data of Cs[Gd(H_2_O)_3_(SO_4_)_2_]·H_2_O are perfectly superimposed on the Brillouin function, whereas a slight deviation is observed for CsGd(SO_4_)_2_. This may be associated with imperfection in the magnetization measurement or with variation in the Gd–Gd distances of these materials (5.1366(19) Å for CsGd(SO_4_)_2_ and 6.4306(6) Å for Cs[Gd(H_2_O)_3_(SO_4_)_2_]·H_2_O). Nevertheless, the subtle deviation of CsGd(SO_4_)_2_ from an ideal overlap with the Brillouin model is not sufficient to override the magnetic isotropy of this material. Derivatives of these *M*(*H*) curves d*M*/d*H* decline quickly and approach zero at μ_0_*H* ≥ 4 T, consistent with the magnetic saturation observed in the *M*(*H*) data. While the Gd and Gd hydrate materials exhibit different structure dimensionality, their magnetic isotropy is preserved. In contrast to certain Gd^3+^ systems possessing exceptional anisotropy with finite orbital magnetic moment, CsGd(SO_4_)_2_ and Cs[Gd(H_2_O)_3_(SO_4_)_2_]·H_2_O maintain the integrity of the isotropic spin-only character of the ^8^*S* ground state. Additional studies such as high-frequency and high-field electron paramagnetic resonance spectroscopy, however, are encouraged to interrogate the small degree of zero-field splitting and the mixing of the ^6^*P* high-lying excited state and the ground state.

### Heat Capacity

**Figure 4:**
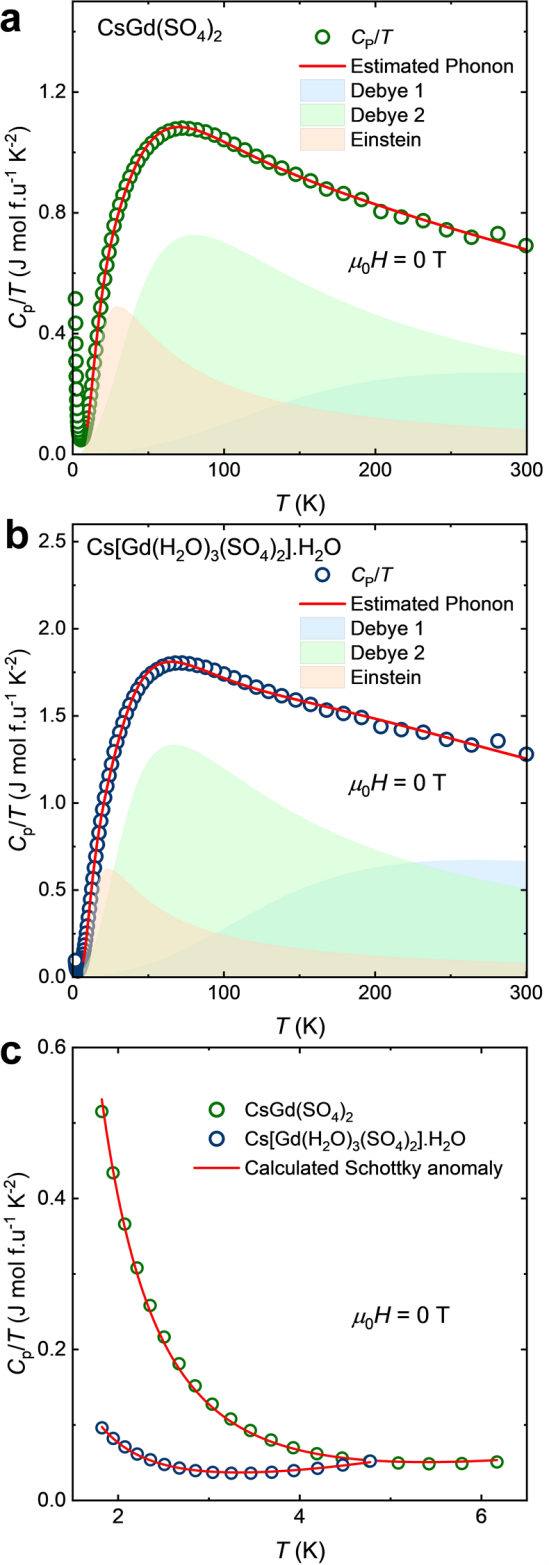
Molar heat capacity over temperature (*C*_P_/*T*) versus temperature (*T*) and phonon analysis for (a) CsGd(SO_4_)_2_ and (b) Cs[Gd(H_2_O)_3_(SO_4_)_2_]·H_2_O. The calculated phonon was best described using a combination of two Debye models and one Einstein model (eq 6). The resulting parameters are presented in Table 2. (c) Analysis of Schottky anomaly.

To study the thermodynamic properties of the ground state of CsGd(SO_4_)_2_ and Cs[Gd(H_2_O)_3_(SO_4_)_2_]·H_2_O, we performed heat capacity measurements at μ_0_*H* = 0 T, 1.8 K ≤ *T* ≤ 300 K. The *C*_P_/*T* versus *T* curves of these Gd materials are presented in Figure 4. As aforementioned, no magnetic phase transition was observed in the magnetization data of the Gd compounds. Thus, their total heat capacity is due to lattice excitations (phonons). The specific heat data were best described using a combination of two Debye and one Einstein modes. Equation 6 shows the fit model, and eqs 7 and 8 represent the Debye and Einstein models, respectively.^41^
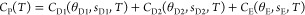
6
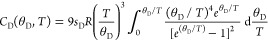
7
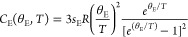
8θ_D1_ and θ_D2_ are the Debye temperatures; θ_E_ is the Einstein temperatures; *s*_D1_, *s*_D2_, and *s*_E_ are the oscillator strengths; and *R* is the molar gas constant. The resulting parameters are presented in Table 2.

**Table 2:** Debye and Einstein Temperatures, θ_D_, θ_E1_, θ_E2_, and Oscillator Strengths, *s*_D_, *s*_E1_, *s*_E2_ Obtained from the Analysis of Phonons in the Molar Heat Capacity over Temperature (*C*_P_/*T*) versus Temperature (*T*) of CsGd(SO_4_)_2_ and Cs[Gd(H_2_O)_3_(SO_4_)_2_]·H_2_O

parameters	CsGd(SO_4_)_2_	Cs[Gd(H_2_O)_3_(SO_4_)_2_]·H_2_O
θ_D1_ (K)	1011(53)	946(31)
θ_D2_ (K)	286(6)	239(5)
θ_E_ (K)	76(1)	61(1)
*s*_D1_	5.40(23)	12.55(37)
*s*_D1_	4.10(11)	6.30(13)
*s*_E_	0.99(4)	1.00(5)

The total numbers of the oscillator strengths are 10.49(38) and 19.85(55) for CsGd(SO_4_)_2_ and Cs[Gd(H_2_O)_3_(SO_4_)_2_]·H_2_O, respectively. These values fall slightly short of the expected oscillator strength of 12 and 24, respectively, which are the number of atoms per formula unit. This is likely due to the coupling of lattice excitations arising from the strong covalent bond in the (SO_4_)^2–^ and H_2_O ligands.

The specific heat data of CsGd(SO_4_)_2_ and Cs[Gd(H_2_O)_3_(SO_4_)_2_]·H_2_O exhibit an upturn at *T* < 4 K, indicating the onset of a Schottky anomaly. This can be attributed to the Gd^3+^ paramagnetic spins.

The *C*_P_/*T* data at *T* ≤ 6 K were analyzed using eq 9 (Figure 4c).^42,43^
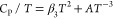
9where β_3_ is the thermal expansion coefficient, which describes the lattice vibration, and *A* is the Schottky parameter which is related to the splitting energy between nondegenerate energy levels. Electronic contribution (γ) was not included in the expression due to the insulating behavior of the compounds. The resulting β_3_ and *A* values for CsGd(SO_4_)_2_ and Cs[Gd(H_2_O)_3_(SO_4_)_2_]·H_2_O obtained from the Schottky fitting are presented in Table 3.

**Table 3:** Fitting Parameters β_3_ (J mol^–1^ K^–4^) and *A* (J K mol^–1^) Obtained from the Schottky Anomalies for CsGd(SO_4_)_2_ and Cs[Gd(H_2_O)_3_(SO_4_)_2_]·H_2_O

parameters	CsGd(SO_4_)_2_	Cs[Gd(H_2_O)_3_(SO_4_)_2_]·H_2_O
β_3_ (J mol^–1^ K^–4^)	0.00105(7)	0.00200(2)
*A* (J K mol^–1^)	3.18(2)	0.548(3)

The estimated β_3_ values of the two compounds are on the same order of magnitude, suggesting similar energy scale for their phonons. It is worth noting that the Schottky estimation is not ideal because only the onset of the anomaly observed down to *T* = 1.8 K was taken into consideration. Nevertheless, the Schottky term suggests that the upturn in the specific heat data is not related to a magnetic phase transition, but rather the Schottky effect associated with the Gd^3+^ paramagnetic systems.

## Conclusion

Despite the isotropic nature of the ^8^*S*_7/2_ ground state in a trivalent gadolinium system, some Gd compounds show a finite orbital angular momentum which is influenced by the crystal field and structural distortion. Keeping the free-ion property of Gd(III) intact even in diverse structural constructs allows design precision and reproducibility of the spin-only nature of 4f^7^ materials, yet this remains unexplored. To address this, we created two new trivalent gadolinium materials exhibiting structurally distinct features. CsGd(SO_4_)_2_ possesses a 2-D structure while Cs[Gd(H_2_O)_3_(SO_4_)_2_]·H_2_O has a 3-D lattice extended by O–H---O hydrogen bonds. The synthesis of these two materials demonstrates a feasible approach for investigating the single-ion behavior of Gd^3+^ in dissimilar structure types. Although the lattice dimensions of the Gd complexes are different, their magnetic properties are similar and driven by the isotropic spin-only nature of the ^8^*S* ground state of the 4f^7^ electron structure. The Gd and Gd hydrate materials exhibit no magnetic phase transition down to *T* = 1.8 K and only a Schottky anomaly owing to the Gd^3+^ paramagnetic spins. Collective lattice excitations observed from heat capacity data are likely connected to the covalent bonds of the (SO_4_)^2–^ and H_2_O groups. This work illustrates a protocol for maintaining the single-ion character of the Gd^3+^ cations while placing the spins in extended lattices with different dimensionalities.

## Supporting Information Available

Additional discussion of crystal structure and optical properties of TAS; crystal structure, ^1^H NMR, ^13^C NMR, mass, and FTIR spectra of TAS; UV–vis–NIR spectra and TGA/DSC of all compounds; *C*_p_/*T*^3^ versus *T* for CsGd(SO_4_)_2_ and Cs[Gd(H_2_O)_3_(SO_4_)_2_]·H_2_O; crystallographic data and calculated bond valence sum for CsGd(SO_4_)_2_ and Cs[Gd(H_2_O)_3_(SO_4_)_2_]·H_2_O (PDF)

## Abbreviations

PTFE: polyfluoroethylene
PXRD: powder X-ray diffraction
ATR-FTIR: attenuated total reflection Fourier transform infrared
NMR: nuclear magnetic resonance
UV–vis–NIR: ultraviolet–visible–near-infrared
PPMS: physical properties measurement system
DC: direct current
TGA: thermogravimetric analysis
DSC: differential scanning calorimetry
SOC: spin–orbit coupling
