# Supplementary material for: Single-Ion Behavior in New 2-D and 3-D Gadolinium 4f7 Materials: CsGd(SO4)2 and Cs[Gd(H2O)3(SO4)2]·H2O
Source: ACS Org Inorg Au. 2022 Sep 4;2(6):502–10. doi: 10.1021/acsorginorgau.2c00031 (PMC9955392; doi:10.1021/acsorginorgau.2c00031)
Supplement: Supplementary file 1 — gg2c00031_si_001.pdf [file gg2c00031_si_001.pdf]

Supporting Information

Single-Ion Behavior in New 2-D and 3-D Gadolinium $4f^7$ Materials: $CsGd(SO_4)_2$ and $Cs[Gd(H_2O)_3(SO_4)_2].H_2O$

Ebube E. Oyeka[1] and Thao T. Tran*,[1]

[1]Department of Chemistry, Clemson University, Clemson, SC 29634, USA

Figure S1. (a) Molecular structure of TAS determined by single-crystal XRD. Ellipsoids are shown at 50 % probability levels.

Figure S2. Packing interactions in the structure of TAS.

Scheme 1. Synthesis of $CsGd(SO_4)_2$ and $Cs[Gd(H_2O)_3(SO_4)_2].H_2O$.

Figure S3. ^{1}H NMR spectra of $C_{12}H_{10}N_2OSSe$ (TAS) (a) full spectrum (b) enlarged scale.

Figure S4. ^{13}C NMR spectra of $C_{12}H_{10}N_2OSSe$ (TAS) (a) full spectrum (b) enlarged scale.

Figure S5. Mass spectra of TAS.

Figure S6. FTIR spectra of $C_{12}H_{10}N_2OSSe$ (TAS) showing characteristic N-H and C=Se stretching vibration peaks at 3264 and 632 cm^{-1}.

Figure S7. UV-Vis-NIR spectra of $C_{12}H_{10}N_2OSSe$ (TAS) presented as a plot of absorbance versus wavelength (nm). The wavelength of maximum absorption is λ_{max} = 294 nm, and the molar absorptivity coefficient at this wavelength was calculated to be $\varepsilon_{\lambda max}$ = 23651 dm^3 mol^{-1} cm^{-1}. The absorption bands at 336 nm and 294 nm were ascribed to $\pi^* \leftarrow n$ electronic transitions of C=Se and C=S bonds respectively.

Figure S8. Thermogravimetric analysis (TGA) and differential scanning calorimetry (DSC) of $C_{12}H_{10}N_2OSSe$ (TAS) showing the melting temperature at 143 °C and thermal decomposition temperature at 174°C followed by a continuous mass loss.

Figure S9. (a,b) Optical absorption spectra for $CsGd(SO_4)_2$ and $Cs[Gd(H_2O)_3(SO_4)_2].H_2O$ respectively showing characteristics electronic transitions. (c) A representation for ground state and first excited state for f^7 (Gd^{3+}) with spin-orbit ($L + S$) coupling.

Figure S10. (a) TG/DSC of $CsGd(SO_4)_2$ showing mass reduction corresponding to the loss of S + O_2 and further at ~925 °C. (b) Thermogravimetry (TG) and differential scanning calorimetry (DSC) of $Cs[Gd(H_2O)_3(SO_4)_2].H_2O$ showing the loss of 4 H_2O molecules at ~265 °C and further decomposition at ~900 °C.

Figure S11. C_p/T^3 versus T for $CsGd(SO_4)_2$. The bump at ~15 K indicates the presence of the Einstein vibration mode.

Figure S12. Molar heat capacity over cubic temperature (C_p/T^3) versus temperature for $Cs[Gd(H_2O)_3(SO_4)_2].H_2O$. The bump at ~12 K indicates the presence of the Einstein vibration mode.

Table S1. Crystallographic data of TAS as obtained by single-crystal XRD.

Table S2. Atomic positions for $CsGd(SO_4)_2$ obtained from single-crystal XRD experiment at T = 297(2) K.

Table S3. Atomic positions for $Cs[Gd(H_2O)_3(SO_4)_2].H_2O$ obtained from single-crystal XRD experiment at T = 297(2) K.

Table S4. Bond lengths (Å) of $CsGd(SO_4)_2$ obtained from single-crystal XRD.

Table S5. Bond lengths (Å) of $Cs[Gd(H_2O)_3(SO_4)_2].H_2O$ obtained from single-crystal XRD.

Table S6. Calculated bond valence sum (V_i) for $CsGd(SO_4)_2$.

Table S7. Calculated bond valence sum (V_i) for $Cs[Gd(H_2O)_3(SO_4)_2].H_2O$.

RESULTS AND DISCUSSION

Characterization of $C_{12}H_{10}N_2OSSe$ (TAS). TAS crystallizes in a monoclinic $P2_1/n$ space group with four molecules occupying a unit cell. The molecule comprises three structural features: a thiophene ring, a phenyl ring, and an acylselenourea moiety. The thiophene and phenyl rings are essentially planar, as supported by ring torsion angles of 0.69 ° and -0.37 °, respectively (Figure S1).

The aromatic rings are linked by the acylselenourea functionality, which provides flexibility to the molecule. The acylselenourea have twisted conformation (torsion angle, -174.52 °) to minimize electronic repulsion between the C=Se and C=O and was geometrically stabilized by C=O---N-H intramolecular H-bond to give a six-membered ring system. The acylselenourea C-N bonds are shorter than the conventional C-N single bond.[1] This is due to the delocalization of electron density between the C=Se and C=O π-electrons and lone-pair electrons on the nitrogen.[1] This delocalization results in acidic NH, proton observed in the ^{1}H NMR spectra at δ 12.83 (Figure S2). The protonated N2 atom of the acylselenourea linker is arranged for an intramolecular interaction with the oxygen atom of the same linker. The protonated N1 atom of the acylselenoura linker is oriented for a weak intermolecular hydrogen bond with the selenium atom of a neighboring molecule, creating a dimer arrangement. The TAS molecules are further stabilized by van der Waals forces and offset π-π stacking interactions of the phenyl groups with the acylselenourea linkers (Figure S1). The optical absorbance of TAS measured in DCM solution at λ = 2500 – 250 nm (Figure S4) shows two absorption bands at 336 nm and 294 nm ascribed to $\pi^*\leftarrow$n electronic transitions of the C=Se and C=S bonds, respectively.

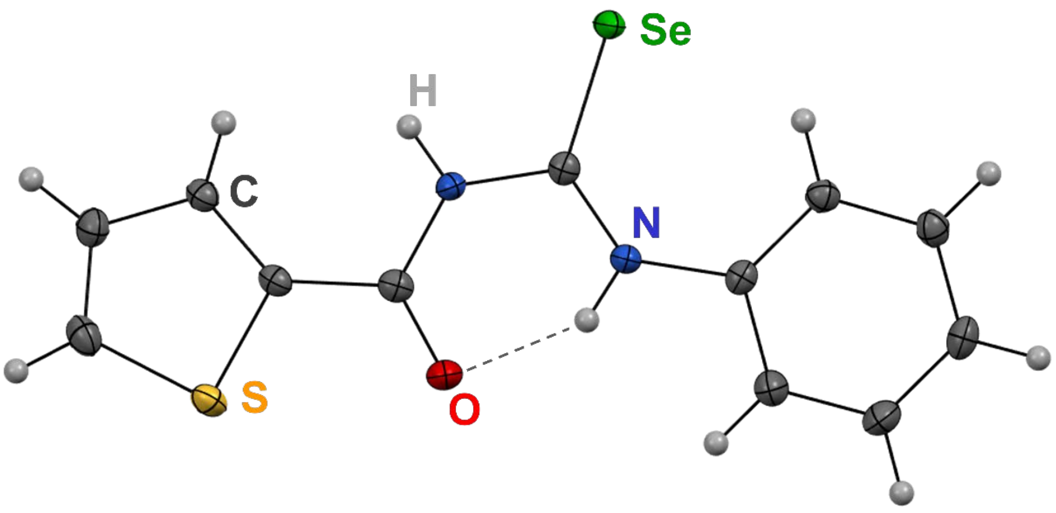

Figure S1. (a) Molecular structure of TAS determined by single-crystal XRD. Ellipsoids are shown at 50 % probability levels.

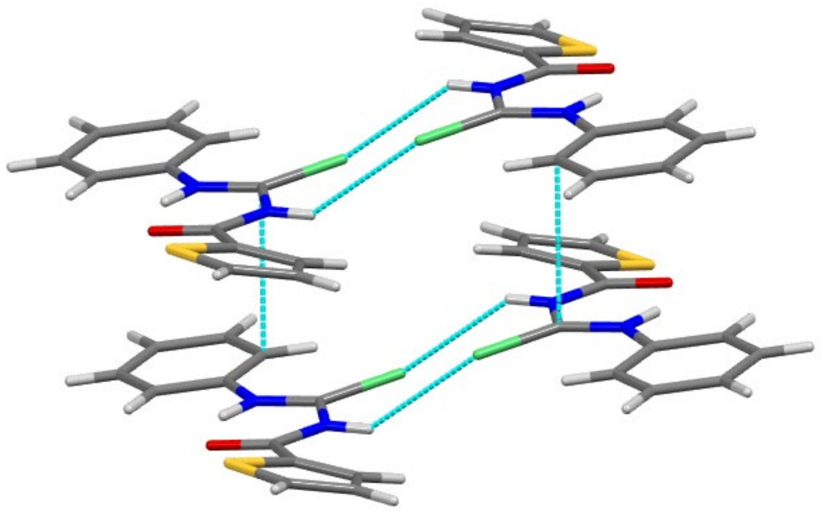

Figure S2. Packing interactions in the structure of TAS.

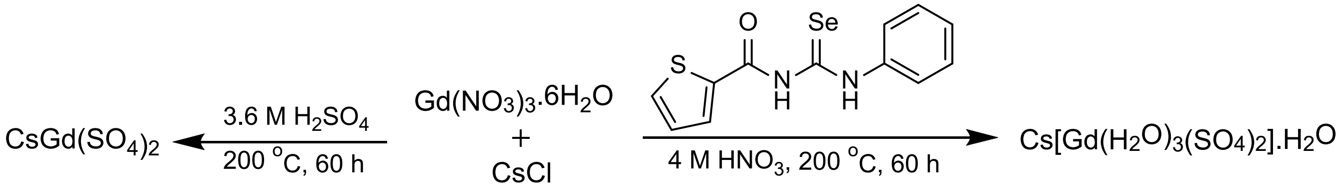

Scheme 1. Synthesis of $CsGd(SO_4)_2$ and $Cs[Gd(H_2O)_3(SO_4)_2].H_2O$.

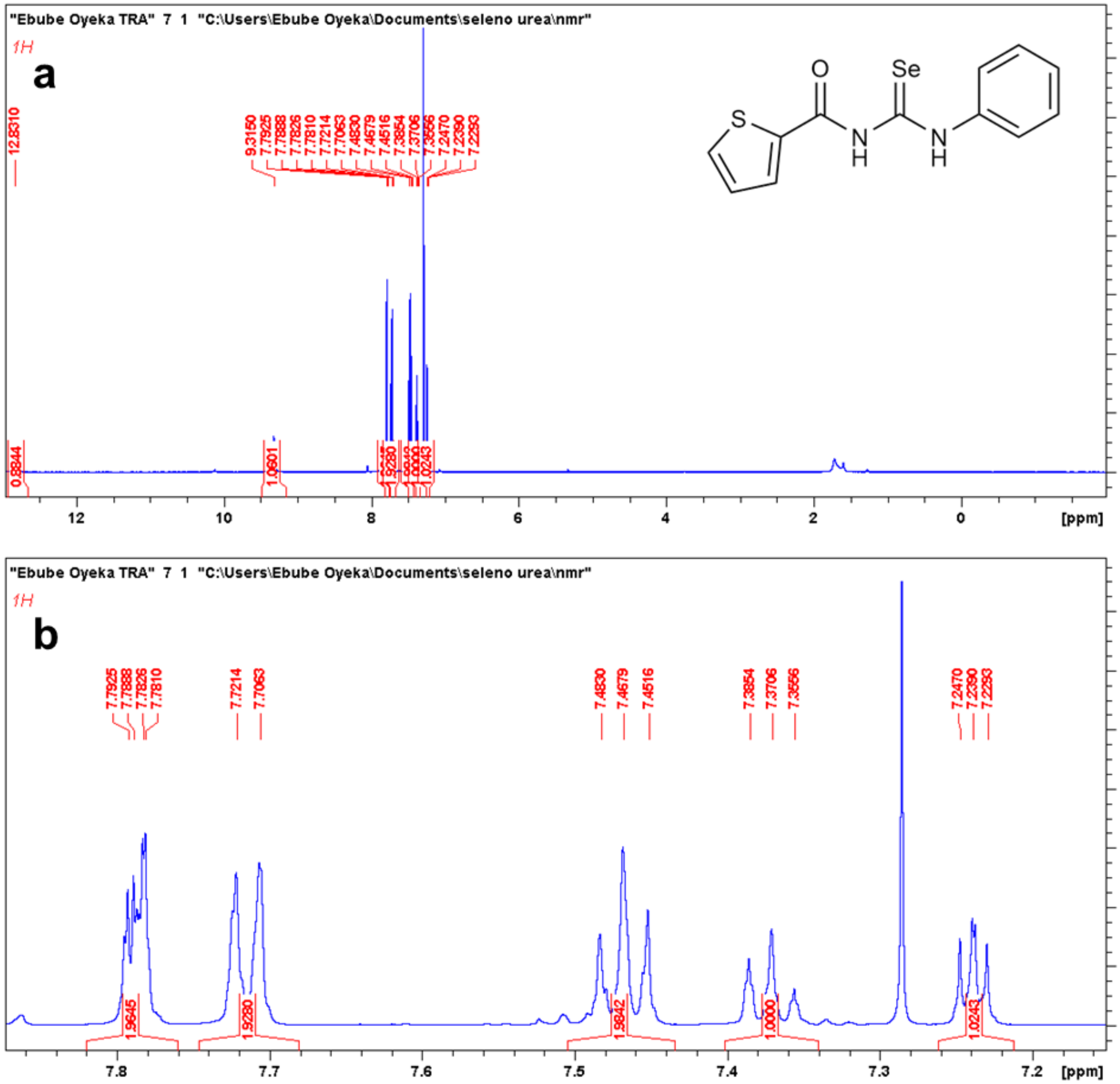

Figure S3. ^{1}H NMR spectra of $C_{12}H_{10}N_2OSSe$ (TAS) (a) full spectrum (b) enlarged scale.

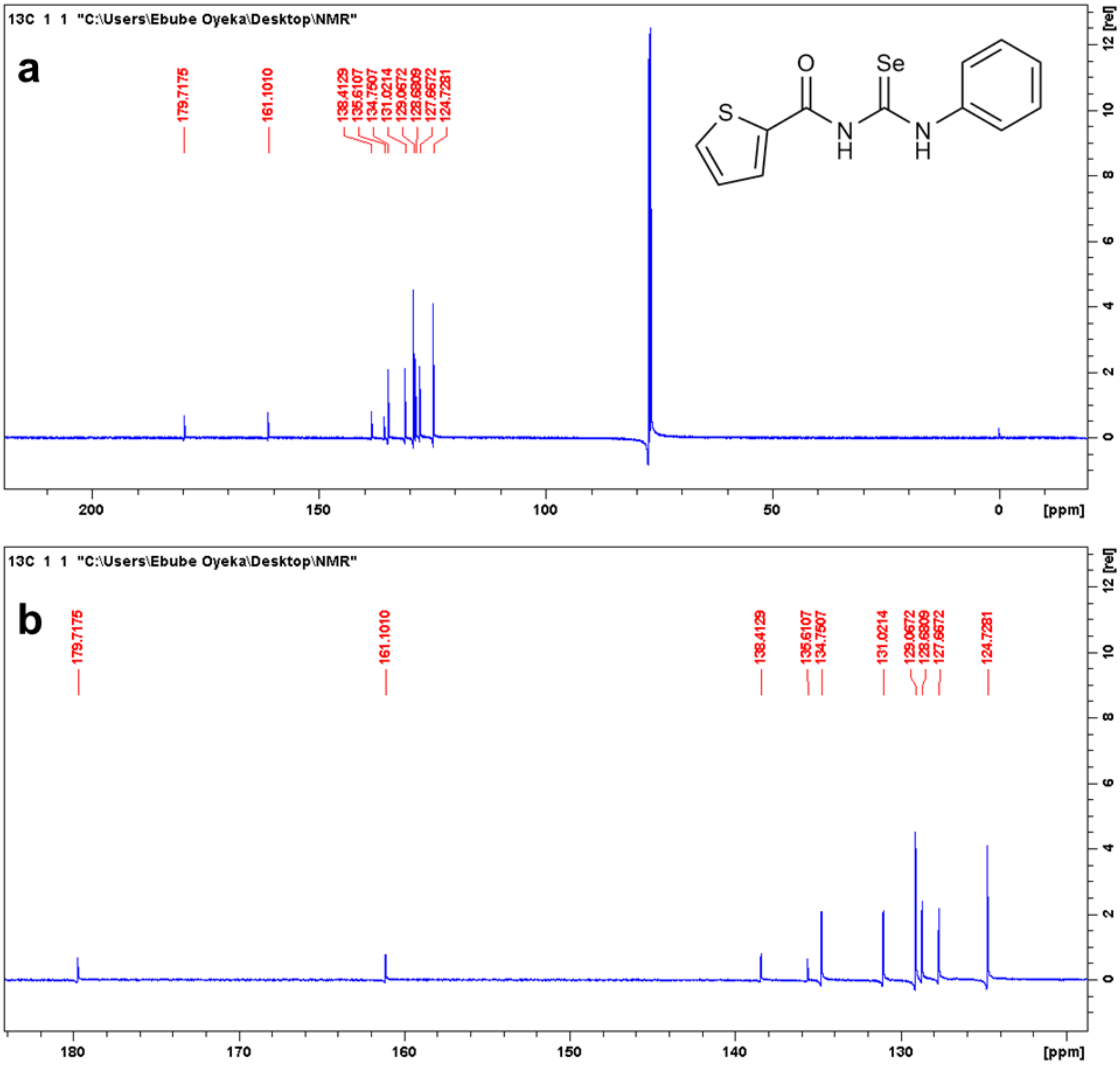

Figure S4. ^{13}C NMR spectra of $C_{12}H_{10}N_2OSSe$ (TAS) (a) full spectrum (b) enlarged scale.

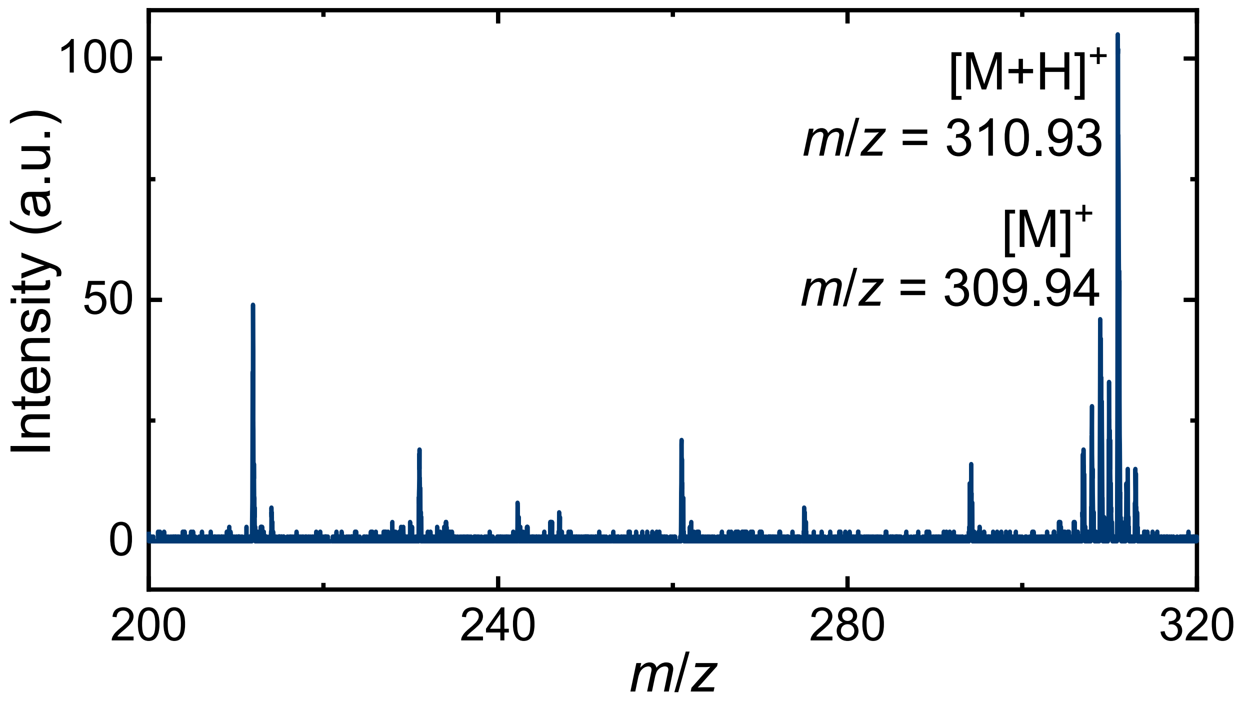

Figure S5. Mass spectra of TAS.

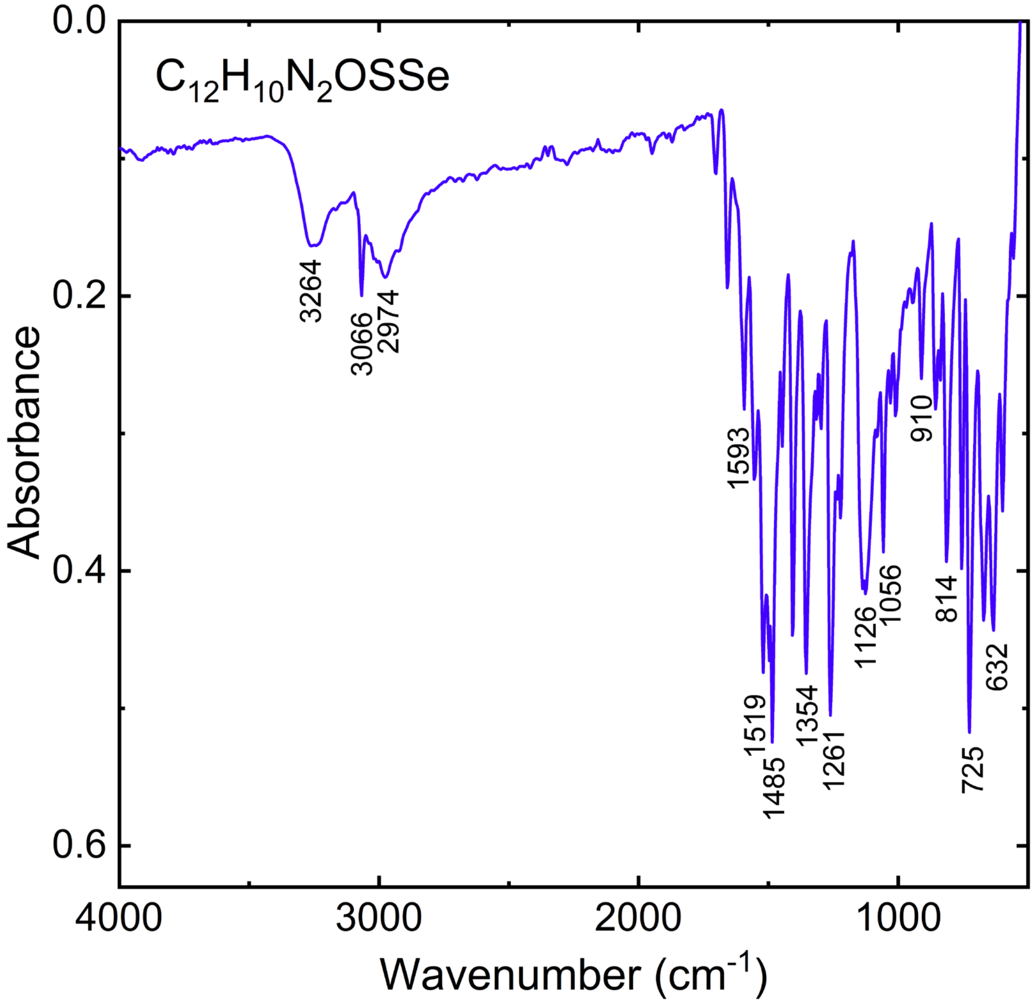

Figure S6. FTIR spectra of $C_{12}H_{10}N_2OSSe$ (TAS) showing characteristic N-H and C=Se stretching vibration peaks at 3264 and 632 cm^{-1}.

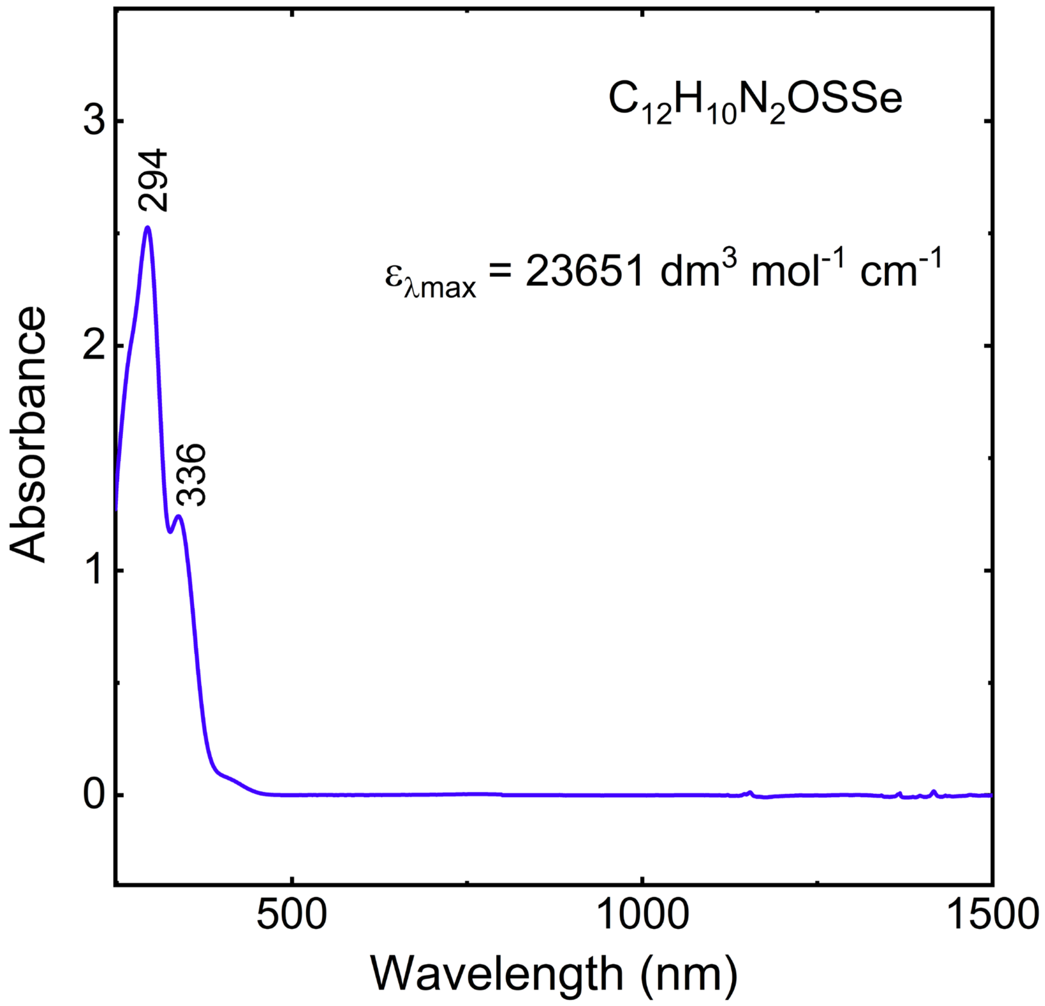

Figure S7. UV-Vis-NIR spectra of $C_{12}H_{10}N_2OSSe$ (TAS) presented as a plot of absorbance versus wavelength (nm). The wavelength of maximum absorption is λ_{max} = 294 nm, and the molar absorptivity coefficient at this wavelength was calculated to be $\varepsilon_{\lambda max}$ = 23651 dm^3 mol^{-1} cm^{-1}. The absorption bands at 336 nm and 294 nm were ascribed to $\pi^*\leftarrow$n electronic transitions of C=Se and C=S bonds respectively.

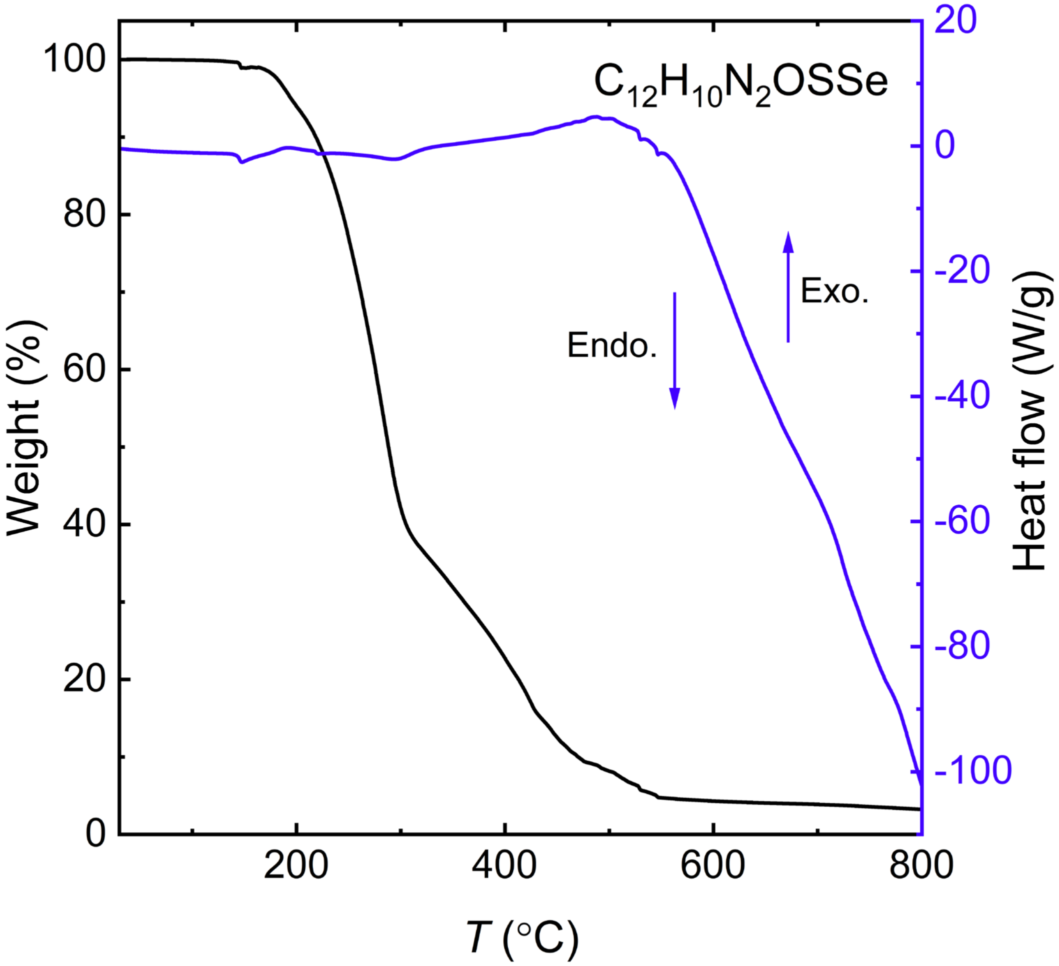

Figure S8. Thermogravimetric analysis (TGA) and differential scanning calorimetry (DSC) of $C_{12}H_{10}N_2OSSe$ (TAS) showing the melting temperature at 143 °C and thermal decomposition temperature at 174°C followed by a continuous mass loss.

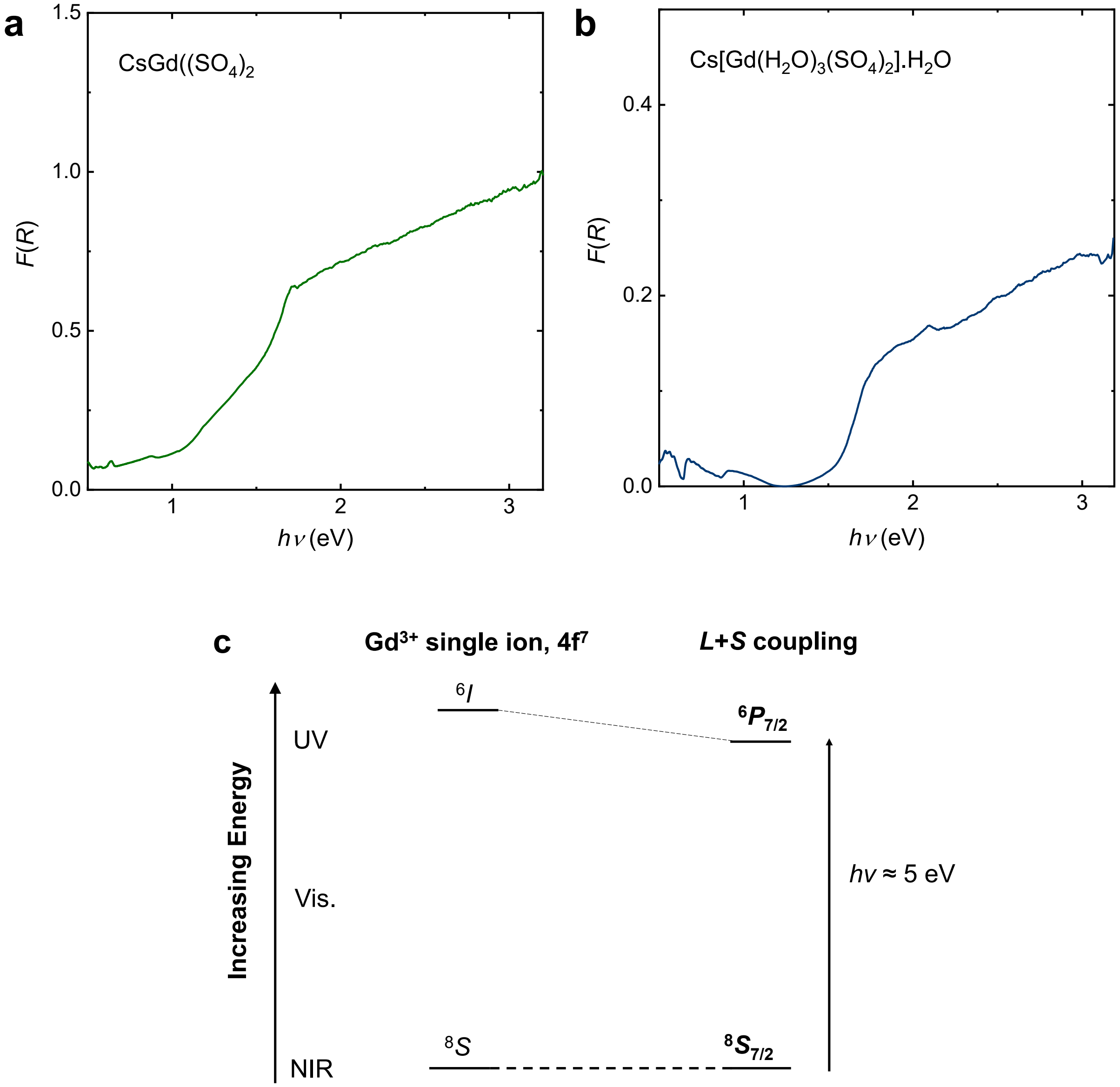

Figure S9. (a,b) Optical absorption spectra for $CsGd(SO_4)_2$ and $Cs[Gd(H_2O)_3(SO_4)_2].H_2O$ respectively showing characteristics electronic transitions. (c) A representation for ground state and first excited state for f^7 (Gd^{3+}) with spin-orbit ($L + S$) coupling.[2]

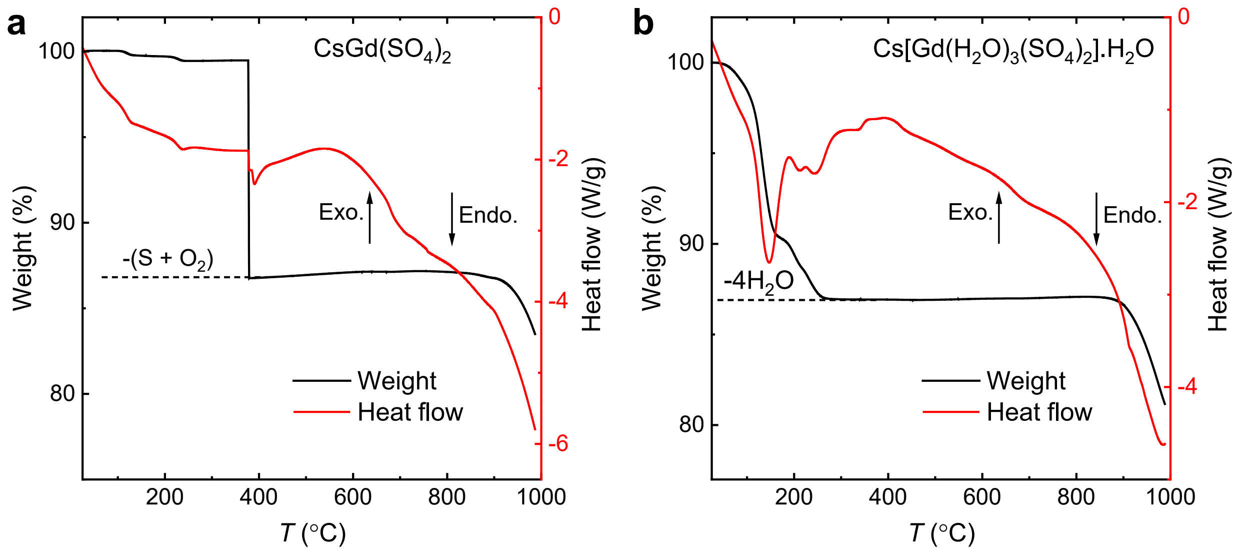

Figure S10. (a) TG/DSC of $CsGd(SO_4)_2$ showing mass reduction corresponding to the loss of S + O_2 and further at ~925 °C. (b) Thermogravimetry (TG) and differential scanning calorimetry (DSC) of $Cs[Gd(H_2O)_3(SO_4)_2].H_2O$ showing the loss of 4 H_2O molecules at ~265 °C and further decomposition at ~900 °C.

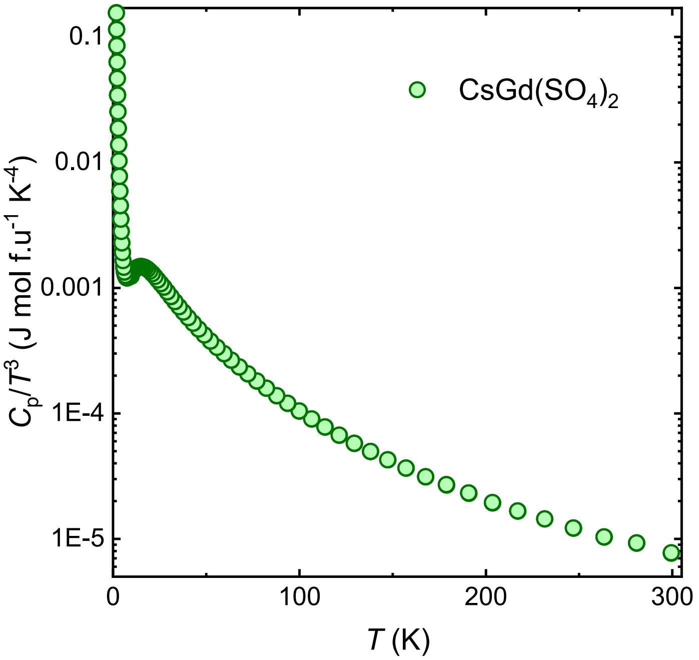

Figure S11. C_p/T^3 versus T for $CsGd(SO_4)_2$. The hump at ~15 K indicates the presence of the Einstein mode.

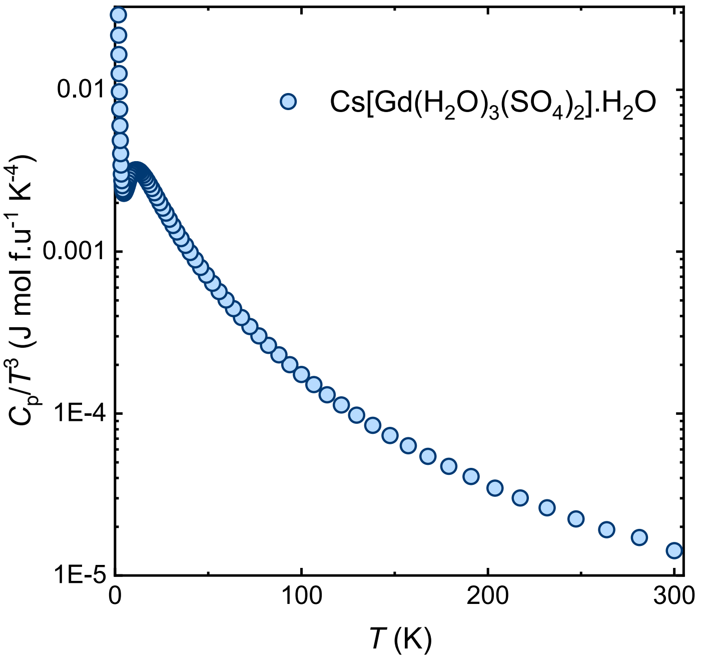

Figure S12. Molar heat capacity over cubic temperature (C_p/T^3) versus temperature for $Cs[Gd(H_2O)_3(SO_4)_2].H_2O$. The hump at ~12 K indicates the presence of the Einstein mode.

Table S1. Crystallographic data of TAS as obtained by single-crystal XRD.

	TAS
Formula	$C_{12}H_{10}N_2OSSe$
M (g mol^{-1})	309.24
T (K)	100
X-ray radiation	Mo$K\alpha$
λ (Å)	0.71073
Crystal system	Monoclinic
Space group	$P2_1/n$
Z	4
a (Å)	9.8776(5)
b (Å)	4.6465(2)
c (Å)	26.6247(13)
β (°)	99.141(2)
V(Å^3)	1206.46(10)
ρ_{calc} (g cm^{-1})	1.703
Reflns collected/unique/obs	16501/ 2745 / 2387
No. of parameters	154
μ (mm^{-1})	3.268
$2\theta_{max}$ (°)	55.0
GOF	1.153
R(F)[a]	0.0247
R_w (F^2)[b]	0.0511
Flack parameter	-

[a] $R(F) = \sum \| F_o| - |F_o^2\| / \sum |F_o|$ (1)

[b] $R_w(F_o^2) = [\sum w\,(F_o^2 - F_o^2)^2 / \sum w(F_o^2)^2]^{1/2}$ (2)

Table S2. Atomic positions for $CsGd(SO_4)_2$ obtained from single-crystal XRD experiment at *T* = 297(2) K.

	x	***y***	***z***	***U*(A²)**
Gd	0.250000	0.500000	0.82254(5)	0.01637(14)
Cs	0.07880(3)	0.250000	0.250000	0.02368(15)
S1	0.41415(8)	0.41804(6)	0.26347(16)	0.0162(2)
O1	0.3476(3)	0.40046(19)	0.5061(5)	0.0204(5)
O2	0.5637(3)	0.3914(2)	0.2797(5)	0.0201(5)
O3	0.3437(3)	0.36266(17)	0.0629(5)	0.0189(5)
O4	0.4003(3)	0.5204(2)	0.1922(5)	0.0207(5)

Table S3. Atomic positions for $Cs[Gd(H_2O)_3(SO_4)_2].H_2O$ obtained from single-crystal XRD experiment at *T* = 297(2) K.

	x	***y***	***z***	***U*(A²)**
Gd	0.81209(2)	0.37274(2)	0.19857(2)	0.01032(6)
Cs	1.32234(4)	0.21650(2)	0.35332(3)	0.02426(8)
S1	0.84002(13)	0.27788(4)	0.56991(9)	0.01253(16)
S2	0.26934(13)	0.42204(5)	0.21361(10)	0.01366(16)
O1	0.8199(5)	0.29378(14)	0.4065(3)	0.0216(6)
O2	0.6506(4)	0.24358(15)	0.6150(3)	0.0185(5)
O3	0.8871(5)	0.34194(14)	0.6572(3)	0.0231(6)
O4	1.0055(4)	0.22515(14)	0.6004(3)	0.0185(5)
O5	0.1271(4)	0.41934(15)	0.0746(3)	0.0200(5)
O6	0.1480(4)	0.38957(17)	0.3333(3)	0.0224(6)
O7	0.4505(4)	0.37814(15)	0.1921(4)	0.0250(6)
O8	0.3279(5)	0.49353(15)	0.2521(4)	0.0264(6)
O9W	0.7681(6)	0.49543(16)	0.1528(4)	0.0328(8)
H9A	0.829(9)	0.518(3)	0.087(6)	0.06(2)
H9B	0.757(15)	0.521(4)	0.231(6)	0.12(4)
O10W	0.3498(6)	0.4126(2)	0.6168(4)	0.0358(8)
H10A	0.460(7)	0.423(5)	0.578(8)	0.10(3)
H10B	0.248(7)	0.407(5)	0.555(7)	0.07(3)
O11W	0.7263(5)	0.38548(16)	-0.0750(3)	0.0209(6)
H11A	0.794(7)	0.364(2)	-0.139(5)	0.025(13)
H11B	0.696(11)	0.4265(18)	-0.110(7)	0.07(2)
O12W	0.7483(6)	0.43639(17)	0.4377(4)	0.0319(7)
H12A	0.813(10)	0.419(3)	0.515(5)	0.06(2)
H12B	0.755(13)	0.4809(11)	0.439(9)	0.08(3)

Table S4. Bond lengths (Å) of $CsGd(SO_4)_2$ obtained from single-crystal XRD.

Gd	O1	O2	O3	O4
	2.383(3)	2.398(3)	2.475(3)	2.465(3)
**S1**	**O1**	**O2**	**O3**	**O4**
	1.470(3)	1.477(3)	1.485(3)	1.482(3)

Table S5. Bond lengths (Å) of $Cs[Gd(H_2O)_3(SO_4)_2].H_2O$ obtained from single-crystal XRD.

Gd	O1	O2	O4	O5	O6	O7	O9W	O11W	O12W
	2.371(3)	2.544(3)	2.445(3)	2.555(3)	2.447(3)	2.363(3)	2.387(3)	2.453(3)	2.493(3)
**S1**	**O1**	**O2**	**O3**	**O4**					
	1.470(3)	1.478(3)	1.465(3)	1.488(3)					
**S2**	**O5**	**O6**	**O7**	**O8**					
	1.490(3)	1.497(3)	1.473(3)	1.450(3)					

Table S6. Calculated bond valence sum (V_i) for $CsGd(SO_4)_2$.[3]

Gd	O1	O2	O3	O4	$V_i = \sum S_{ij}$
R_{ij}	2.383(3)	2.398(3)	2.475(3)	2.465(3)	
S_{ij}	0.423	0.407	0.330	0.339	2.998
**S1**	**O1**	**O2**	**O3**	**O4**	
R_{ij}	1.470(3)	1.477(3)	1.485(3)	1.482(3)	
S_{ij}	1.516	1.488	1.456	1.468	5.928

Table S7. Calculated bond valence sum (V_i) for $Cs[Gd(H_2O)_3(SO_4)_2].H_2O$.[3]

Gd	O1	O2	O4	O5	O6	O7	O9W	O11W	O12W	$V_i = \sum S_{ij}$
R_{ij}	2.371(3)	2.544(3)	2.445(3)	2.555(3)	2.447(3)	2.363(3)	2.387(3)	2.453(3)	2.493(3)	
S_{ij}	0.437	0.274	0.358	0.266	0.356	0.447	0.419	0.350	0.315	3.222
**S1**	**O1**	**O2**	**O3**	**O4**						
R_{ij}	1.470(3)	1.478(3)	1.465(3)	1.488(3)						
S_{ij}	1.516	1.484	1.537	1.444						5.923
**S2**	**O5**	**O6**	**O7**	**O8**						
R_{ij}	1.490(3)	1.497(3)	1.473(3)	1.450(3)						
S_{ij}	1.436	1.410	1.504	1.600						5.950

References

1. Oyeka, E. E.; Babahan, I.; Eboma, B.; Ifeanyieze, K. J.; Okpareke, O. C.; Coban, E. P.; Özmen, A.; Coban, B.; Aksel, M.; Özdemir, N.; Groutso, T. V.; Ayogu, J. I.; Yildiz, U.; Dinçer Bilgin, M.; Halil Biyik, H.; Schrage, B. R.; Ziegler, C. J.; Asegbeloyin, J. N., Biologically active acylthioureas and their Ni(II) and Cu(II) Complexes: Structural, spectroscopic, anti-proliferative, nucleolytic and antimicrobial studies. *Inorg. Chim. Acta* **2021,** *528*, 120590.

2. Bünzli, J.-C. G., Benefiting from the unique properties of lanthanide ions. *Acc. Chem. Res.* **2006,** *39* (1), 53-61.

3. Altermatt, D.; Brown, I. D., The automatic searching for chemical bonds in inorganic crystal structures. *Acta Crystallogr. B* **1985,** *41* (4), 240-244.
